# Anterior Segment Optical Coherence Tomography for the Tailored Treatment of Mooren’s Ulcer: A Case Report

**DOI:** 10.3390/jcm13185384

**Published:** 2024-09-11

**Authors:** Luca Lucchino, Elvia Mastrogiuseppe, Francesca Giovannetti, Alice Bruscolini, Marco Marenco, Alessandro Lambiase

**Affiliations:** Department of Sense Organs, Sapienza University of Rome, Viale del Policlinico 155, 00161 Rome, Italy; luca.lucchino@uniroma1.it (L.L.); elvia.mastrogiuseppe@uniroma1.it (E.M.); alice.bruscolini@uniroma1.it (A.B.); marco.marenco@uniroma1.it (M.M.); alessandro.lambiase@uniroma1.it (A.L.)

**Keywords:** Mooren’s ulcer, AS-OCT, PUK, cyclosporine A, corneal imaging

## Abstract

**Background:** Mooren’s ulcer (MU) is a rare and debilitating form of peripheral ulcerative keratitis (PUK), characterized by a crescent-shaped ulcer with a distinctive overhanging edge at the corneal periphery. If left untreated, MU can lead to severe complications such as corneal perforation and blindness. Despite various treatment approaches, including anti-inflammatory and cytotoxic drugs, as well as surgical interventions, there is no clear evidence of the most effective treatment due to the lack of randomized controlled trials. AS-OCT is a non-invasive imaging technique that provides high-resolution cross-sectional images of the anterior segment, allowing for accurate evaluation of corneal ulcer characteristics, including depth, extent, and disease progression. **Methods:** We present the case of a 20-year-old male patient with MU managed using a stepladder approach, which included local and systemic corticosteroids, limbal conjunctival resection, and Cyclosporine A 1% eye drops. The patient underwent consecutive AS-OCT examinations and strict follow-up to tailor systemic and topical therapy. **Results:** Complete healing of the corneal ulcer with resolution of the inflammatory process was achieved. There was no recurrence of the disease at the 7-month follow-up. AS-OCT demonstrated progressive reorganization and thickening of the stromal tissue until the complete recovery of stromal thickness. **Conclusions:** The AS-OCT imaging modality allowed for the accurate evaluation of corneal ulcer characteristics, facilitating informed decision-making regarding the use of systemic immunosuppression, surgical interventions, and local immunomodulation and providing detailed and precise assessment of disease progression. This approach enabled a tailored and effective treatment strategy for the patient and played a critical role in guiding the therapeutic approach.

## 1. Introduction

Mooren’s ulcer (MU) is a rare and debilitating form of peripheral ulcerative keratitis (PUK), prevalent in southern and central Africa as well as the Indian subcontinent, exhibiting a male predilection [1]. Characterized by moderate to severe pain, MU begins with a crescent-shaped ulcer featuring a distinctive overhanging edge at the corneal periphery. It progresses both centrally and circumferentially with minimal scleral involvement [2].

The pathogenesis of MU involves complex interactions between genetic predisposition, altered immune responses, and environmental factors. Despite ongoing research, the exact etiology of MU remains elusive, and it continues to be a diagnosis of exclusion, posing significant challenges in its management [3,4]. It has been hypothesized that the etiology of MU may result from an autoimmune response to calgranulin C, an antigen typically concealed but expressed by stromal keratinocytes [5]. Sensitization to calgranulin C can be triggered by corneal trauma, infection, or aging-related changes, particularly in subjects with specific haplotype expression (HLA-DR17 and DQ2). This may lead to the activation of antigen-presenting cells, likely stimulating T-cell response and subsequently contributing to ulcer formation. Alternatively, helminthic antigens could potentially induce a similar immune response. Furthermore, complement activation and the release of collagenases by neutrophils are implicated in corneal stromal destruction, resulting in progressive corneal damage [3,6].

Different classifications exist for MU, highlighting that while older subjects typically present with a unilateral and indolent form, younger patients often experience bilateral, more severe disease with poorer treatment outcomes [2,7]. MU can lead to severe complications, such as corneal perforation and blindness if not promptly treated. Various treatments are utilized for MU, including anti-inflammatory drugs (steroidal and non-steroidal), cytotoxic drugs (topical and systemic), conjunctivectomy, and cornea debridement (superficial keratectomy) [8]. Despite advances in understanding MU, it still causes significant ocular morbidity, prompting the use of new biological medications to control inflammation in cases unresponsive to conventional therapies such as TNF-α blockers [9], monoclonal antibody targeting CD20 on B cells [10], and interferon alfa-2a [11]. However, due to the absence of randomized controlled trials (RCTs), there is no clear evidence on the most effective treatment. Therefore, a stepladder approach is typically employed based on clinical judgment and patient characteristics [8].

Anterior Segment Optical Coherence Tomography (AS-OCT) is a non-invasive imaging modality that provides high-resolution cross-sectional images of the eye’s anterior segment using low-coherence interferometry [12]. In corneal pathologies, AS-OCT plays a pivotal role, adjunct to slit lamp biomicroscopy, in diagnosing and managing various conditions. It aids in evaluating corneal opacities, infections, dystrophies, ulcers, and other disorders by offering precise structural information [13]. AS-OCT assesses corneal thickness, identifies scarring or haze, detects synechiae, and assists in planning surgical interventions like keratoplasty. Moreover, it facilitates monitoring disease progression, grading severity, evaluating treatment response, and providing accurate real-time therapeutic guidance [12].

## 2. Case Presentation

**Figure 1:**
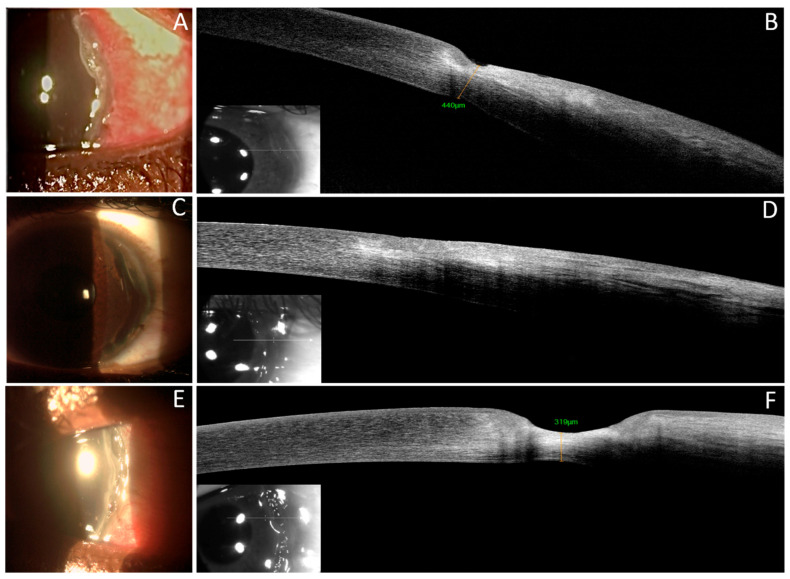
Slit lamp examination and corresponding AS-OCT section pointed out by the line across the area of interest. May (**A**,**B**). Slit lamp biomicroscopy (**A**) shows conjunctival hyperaemia, intense perikeratic reaction, circular paralimbal temporal thinning, and an epithelial defect. AS-OCT scan (**B**) reveals the absence of the epithelium, stromal thinning (residual stromal thickness of 440 μm), and stromal hyper-reflectivity. June 2023 (**C**,**D**): Resolution of the inflammatory condition was observed. The conjunctiva was normoemic with no perikeratic reaction, and the epithelial defect had been repaired (**C**). AS-OCT scan showed that the epithelial layer was irregular and hyporeflective but intact, filling the area of corneal thinning. The underlying stroma was hyper-reflective (**D**). July 2023 (**E**,**F**): Worsening of the clinical condition was observed. Severe perikeratic reaction with temporal ulceration and thinning (**E**). AS-OCT scan revealed the absence of the epithelial layer, significant stromal thinning (319 μm), and stromal hyper-reflectivity (**F**).

A 20-year-old male patient of Senegalese descent presented to our clinic in May 2023 complaining of redness and pain in his left eye (LE) for the past few weeks. The patient’s ocular and general medical history was unremarkable. Upon initial clinical examination, the best corrected visual acuity (BCVA) was 0 Log MAR in both eyes. The right eye showed no pathological signs, but the LE exhibited an intense perikeratic reaction, more pronounced in the temporal sectors, along with a crescent-shaped peripheral corneal ulcer featuring an epithelial defect with an overhanging edge and stromal thinning in the temporal periphery (Figure 1). Intraocular pressure and the rest of the ocular structures were within normal limits. The ongoing topical antibiotics were discontinued for a 48 h washout period, and corneal scraping was performed to rule out infectious keratitis. Once corneal scraping revealed no pathogenic microorganisms, treatment was adjusted to include topical corticosteroids, dexamethasone 1.5 mg/mL drops, administered six times per day, and topical antibiotic coverage with moxifloxacin 3 mg/mL drops four times daily. To promptly identify and manage potential corticosteroid-related complications, such as infection or intraocular pressure elevation, the patient was closely monitored with almost daily examinations.

Comprehensive laboratory investigations were performed, including complete blood count (CBC) with differential, platelet count, erythrocyte sedimentation rate (ESR), rheumatoid factor (RF), anti-cyclic citrullinated peptide (anti-CCP) antibodies, complement fixation test, antinuclear antibodies (ANA), anti-neutrophil cytoplasmic antibodies (ANCA), circulating immune complexes assay, liver function tests, Venereal Disease Research Laboratory (VDRL) or Fluorescent Treponemal Antibody Absorption (FTA-ABS) test, blood urea nitrogen (BUN) and creatinine levels, serum protein electrophoresis, and urinalysis. Additionally, serological tests for Hepatitis C Virus (HCV), Hepatitis B Virus (HBV), Angiotensin-Converting Enzyme (ACE), and QuantiFERON gold test were conducted, along with chest X-ray and rheumatology consultation. After negative findings from all laboratory and instrumental tests for concurrent systemic diseases, as well as rheumatological evaluation, the diagnosis of Mooren’s ulcer was established. Then, after one month, given the patient’s stable condition and the healing of the epithelial defect, topical corticosteroids were gradually tapered for three weeks (Figure 1). 

**Figure 2:**
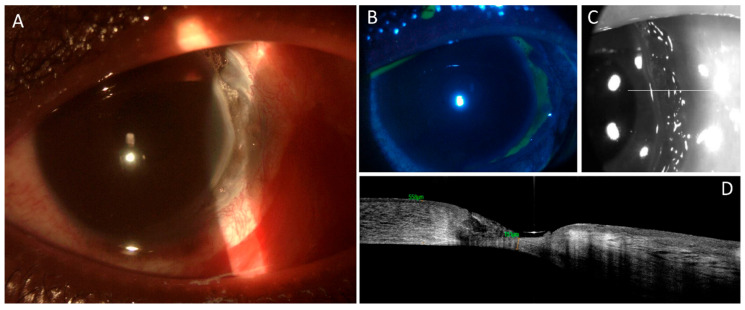
Slit lamp examination (**A**,**B**) and AS-OCT (**C**,**D**) in August 2023. Slit lamp biomicroscopy shows (**A**) conjunctival hyperemia, severe perikeratic reaction, significant circular, paralimbal, temporal thinning, and crescent-shaped ulceration. Corneal ulcer positive on fluorescein vital coloration (**B**). AS-OCT images: line across the section of thinning (**C**). Marked thinning (residual stromal thickness 151 μm), absence of epithelium, pronounced stromal disarray, hyporeflective voids amidst hyper-reflective regions.

The patient was strictly monitored with ocular examinations and AS-OCT imaging, which aided in managing therapy tapering. Corneal thickness measurements were taken using Optovue iVue80 Spectral Domain-OCT (Optovue Inc., Fremont, CA, USA) with the Cornea Anterior Module (CAM), which includes a lens adapter attached to the front of the instrument for imaging the cornea and anterior chamber. AS-OCT scans were performed using the Cornea Angle module, which utilizes a single 5 mm scan line (1 × 1024 A-scans per frame, 16 averaged scans per line) with speckle noise reduction, and a depth resolution of 5 μm. Specifically, linear measurements were recorded at the thinnest point of the cornea with the caliper perpendicular to the endothelium and repeated at the same location during each follow-up visit using the same protocol. The cornea specialist (AL) visually confirmed the measurement location on the infrared images provided by the AS-OCT device to ensure consistency. Unfortunately, at the end of July, the patient returned to our clinic with worsening symptoms, including a severe perikeratic reaction, recurrence of the corneal ulcer, and marked stromal thinning (Figure 1). Therefore, urgent excision of the limbal conjunctiva and tenectomy was performed the next day. Systemic immunosuppression was also started with oral prednisolone, initially 50 mg daily, which was then tapered to 25 mg per day. Local inflammation was managed with peribulbar injections of triamcinolone acetonide (40 mg/mL) and antibiotic coverage was provided with moxifloxacin 3 mg/mL eye drops, administered four times daily in the LE. Despite initial improvement, after two weeks of aggressive therapy, the patient’s condition worsened (Figure 2).

**Figure 3:**
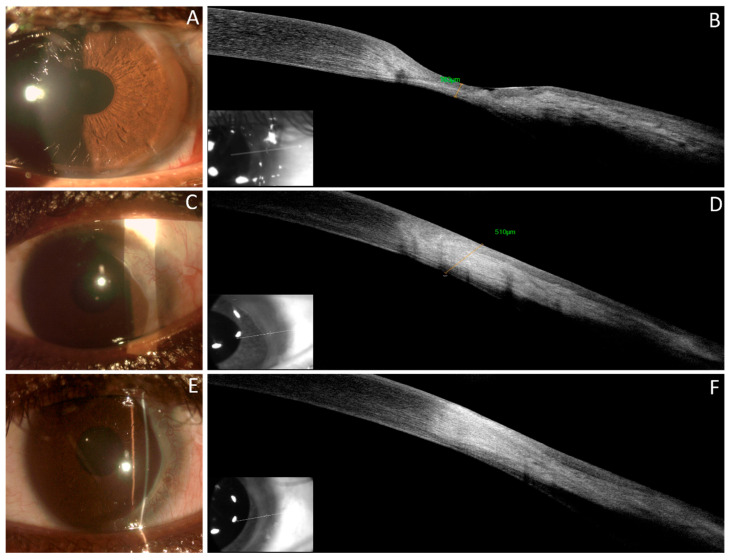
Slit lamp examination and corresponding AS-OCT section pointed out by the line across the area of interest. September (**A**,**B**): At the 1-month follow-up, with topical and systemic therapy, slit lamp biomicroscopy shows reduced conjunctival hyperemia, decreased temporal thinning, and epithelial integrity with initial conjunctivalization (**A**). AS-OCT scans reveal a hyporeflective epithelial layer, significant stromal thinning (residual stromal thickness 163 μm), and diffuse stromal hyper-reflectivity absence of hyporeflective voids. October (**C**,**D**): Resolution of the inflammatory process. Presence of fibrovascular tissue covering the temporal sector (**C**). AS-OCT images demonstrate restored stromal thickness (measured 510 μm) with hyper-reflectivity, indicating substantial stromal remodeling (**D**). December (**E**,**F**). Absence of inflammation (**E**). Enhanced regularity of stromal hyper-reflectivity, suggesting improved alignment of stromal lamellae and overall better tissue organization (**F**).

Therefore, 1% cyclosporine eye drops twice daily were added to the treatment regimen. In September, the resolution of the epithelial defect, with initial conjunctivalization and neovascularization of the tissue, along with early stromal thickening was documented by AS-OCT (Figure 3).

From October to December, oral steroids were slowly tapered while monitoring stromal thickness and continuing topical 1% cyclosporine drops twice daily. AS-OCT revealed progressive reorganization and thickening of the stromal tissue until stromal thickness complete recovery in December (Figure 3). Currently, the patient continues 1% cyclosporine eye drops without recurrence.

## 3. Discussion

PUK can arise from various local or systemic factors, including infectious and noninfectious origins. Accurate identification of the underlying cause is crucial for effective management, necessitating a thorough clinical evaluation, encompassing diverse laboratory and radiological assessments. The diagnosis of MU is a diagnosis of exclusion and requires the absence of an underlying disease [4]. 

As stated in the introduction, controlling inflammation is crucial to prevent the progression of the condition. There are four prevalent strategies to achieve this: local immunosuppression, systemic immunosuppression, removal of the source (limbus), and removal of the target (keratinocytes) [14].

At present, there is a lack of clear evidence regarding treatment selection due to the absence of randomized clinical trials. However, it is suggested to initiate treatment with topical corticosteroids, followed by limbal conjunctival resection when inflammation remains uncontrolled [8].

The excision of the limbal conjunctiva along with tenectomy aims to distance the limbal blood vessels, removing the source and preventing the delivery of antibodies, immune complexes, and metalloproteinases directed against the cornea [15]. Indeed, initial corneal surgery is generally discouraged, while limbal conjunctival resection, apart from being a more conservative approach compared to keratoplasty, has also demonstrated superior disease control when used alongside keratoplasty [16].

In our case, limbal conjunctival resection and systemic immunosuppression with oral steroids did not achieve complete control of inflammation; therefore, after witnessing the progressive worsening of the condition, detected by AS-OCT images, we added to the treatment regimen topical Cyclosporine A 1 mg/mL for two times a day. 

Cyclosporine A (CsA) is a medication used to reduce ocular inflammation. It enters T lymphocytes and forms complexes with intracellular binding proteins. Specifically, this complex inhibits calcineurin phosphatase, disrupting the activation of the NFATc transcription factor and preventing the production of cytokines, including IL-2 and IFN-γ [17]. While primarily studied and used in the treatment of dry eye disease, CsA has also shown efficacy in managing refractory MU [18,19]. In the literature, MU showed a positive response to topical 0.5% cyclosporine in 11 out of 18 cases (61.1%) during long-term follow-up (24–31 months) [18]. Additionally, a reduction in recurrence rates of the condition when combined with keratoplasty at concentrations of 1% and 2% has been demonstrated [16,19].

AS-OCT has proven to be a valuable tool for monitoring the progression and healing of corneal ulcers. Technology has evolved in recent years, enabling detailed assessment of anterior segment structures with finer than slit lamp biomicroscopy [13]. This non-invasive imaging technique allows clinicians to assess various aspects of the ulcer, including the depth of stromal thinning, the extent of the epithelial defect, and the restoration of normal corneal thickness during the healing process. Additionally, AS-OCT provides insights into the structural organization of the cornea, enabling the evaluation of the quality of the scarring and the overall integrity of the tissue [20]. 

Another great advantage of AS-OCT is the ability to provide reliable and reproducible measurements in case of stromal opacities, whereas corneal topographers, owing to scattered light from corneal opacities, struggle to accurately measure eyes with severe opacities [12]. Yoshihara et al. used three-dimensional AS-OCT to evaluate corneal shape, revealing that in eyes with MU, irregular astigmatism and distortion increase as the lesion moves closer to the center [21]. Our patient maintained 20/20 visual acuity by controlling inflammation and keeping the lesion peripheric. 

AS-OCT is recommended for preoperative evaluation in surgical planning before keratoplasty, as it provides precise measurements of the depth of stromal opacities, which is a key determinant for predicting the success of lamellar keratoplasty [22]. Lian et al. performed therapeutic lamellar keratoplasty on six eyes of patients with Mooren’s ulcer. The surgical procedure was optimized by AS-OCT precise measurements of stromal thickness beneath areas of fibrovascular tissue that potentially revealed occult perforations during surgical planning. Additionally, AS-OCT enabled postoperative evaluation of the posterior corneal surface to detect any ectatic areas [23].

In the literature, AS-OCT has been utilized for monitoring disease activity and categorizing PUK in patients affected by vasculitis into three stages: (1) acute stage; (2) healing stage; and (3) healed stage. During the acute phase, AS-OCT typically reveals the absence of corneal epithelium and disorganized anterior stroma with focal thinning and heterogeneous stromal reflectivity. In the healing stage, AS-OCT images often exhibit an irregular epithelium with reduced reflectivity and a less heterogeneous stroma. Finally, in the healed stage, AS-OCT reveals a hyporeflective irregular epithelium, a demarcation line between the hyporeflective epithelium, and hyper-reflective stroma [24].

To our knowledge, this represents the first case in the literature of monitoring MU using AS-OCT. Compared to descriptions of PUK secondary to peripheral vasculitis, our case exhibited distinctive characteristics during the three stages. In the acute phases, in addition to the absence of epithelium and irregular appearance of the stroma, pronounced stromal disarray was observed, featuring hyporeflective voids amidst hyper-reflective regions, indicative of stromal edema in the context of significant inflammation. Later, during the healing process, we observed complete restoration of stromal thickness accompanied by stromal tissue reorganization and diminished hyper-reflectivity in the fully healed phase, indicative of significant stromal remodeling. The hyper-reflectivity may indicate variations in refractive indices or reflective properties of the remodeled corneal stroma. This allowed for tailored therapy of the different stages of the corneal ulcer and, finally, for a full restoration of corneal integrity. This case report aims to highlight the importance of AS-OCT guidance in managing patients affected by complex inflammatory processes such as MU, reminding ophthalmologists to incorporate this essential tool in their clinical practice. While a cornea specialist can easily diagnose ulcerative keratitis, AS-OCT is crucial to provide exact micron measurements, teamwork with colleagues with easy follow-up, and accurate detection of signs of disease progression. Diagnostic guidelines are needed to universalize AS-OCT patterns of inflammation.

## 4. Conclusions

In conclusion, our case underscores the importance of early diagnosis and personalized management in MU. AS-OCT has proven crucial in evaluating disease progression and guiding treatment by accurate detection of disease activity patterns. A tailored therapeutic approach, incorporating systemic immunosuppression with corticosteroids, surgical intervention with limbal conjunctival resection, and local immunomodulation using 1% CsA eye drops, was employed to address inflammation and promote healing, allowing for resolution after four months of treatment.

## Data Availability

Data is available upon reasonable request.
